# Periostin: Immunomodulatory Effects on Oral Diseases

**DOI:** 10.1055/s-0040-1714037

**Published:** 2020-07-20

**Authors:** Zohaib Khurshid, Maria Mali, Necdet Adanir, Muhammad Sohail Zafar, Rabia Sannam Khan, Muhammad Latif

**Affiliations:** 1 Department of Prosthodontics and Dental Implantology, College of Dentistry, King Faisal University, Al Ahsa, Saudi Arabia; 2 Department of Orthodontics, Islamic International Dental College, Riphah International University, Islamabad, Pakistan; 3 Department of Restorative Dentistry, College of Dentistry, King Faisal University, Al Ahsa, Saudi Arabia; 4 Department of Restorative Dentistry, College of Dentistry, Taibah University, Madinah Al-Munawarah, Madinah, Saudi Arabia; 5 Department of Dental Materials, Islamic International Dental College, Riphah International University, Islamabad, Pakistan; 6 Department of Bioengineering, Lancaster University, Lancaster, United Kingdom; 7 Centre for Genetics and Inherited Diseases, College of Medicine, Taibah University, Madinah Al-Munawarah, Madinah, Saudi Arabia

**Keywords:** biomarkers, oral, health, periostin and periodontitis

## Abstract

Periostin is a microcellular adapter protein. It plays a wide range of essential roles during the development and in immunomodulation. Periostin is a prominent contributor during the process of angiogenesis, tumorigenesis, and cardiac repair. It is expressed in periodontal ligaments, tendons, skin, adipose tissues, muscle, and bone. This is a protein-based biomolecule that has the diagnostic and monitoring capability and can potentially be used as a biomarker to detect physiological and pathological conditions. The aim of the present review was to explore the periostin morphology and associated structural features. Additionally, periostin’s immunomodulatory effects and associated biomarkers in context of oral diseases have been discussed.

## Introduction

The periodontal disease is a known inflammatory disease caused by bacteria that destroy the supporting tissue, that is, periodontium. 1 The disease is highly complex affecting approximately 11.2% and around 743 million people were affected. 2 The periodontium is highly enriched with the cellular and molecular components. 3 4 Cellular components include osteoblast, cementoblast, sensory cells, endothelial cells, undifferentiated mesenchymal cells, progenitor stem cells, epithelial rests cells of Malassez, macrophages, fibroblasts, and osteoclasts. 5 The molecular components comprise various extracellular matrix (ECM) proteins, collagen fibers, chemical mediators, glycosaminoglycan, glycoproteins, and glycolipids. One of the essential molecular elements that are highly expressed within periodontal ligament (PDL) is periostin proteins useful for orthodontic tooth movement. Modification of immune response through therapeutic interventions is known as immunomodulation. Several immunomodulatory techniques are available such as vaccines against infections for its prevention.

### Periostin, a Novel Protein

Periostin is found in collagen-rich tissues by continuous mechanical stresses and highly expressed in extracellular protein matrix. 6 Matricellular proteins imply heterogeneous group proteins are present within ECM while interacts either with other cell matrix proteins or with receptors present on the surface of the cells, cytokines, and growth factors. There are many matricellular proteins, that is, bone sialoproteins, CCN2, Cyr61, tenascin-C, periostin, galectin (1, 2, 3, 4, 8, and 9), osteopontin, and osteonectin. All of them are very important in tissue remodeling and wound repair in adult tissues. 7 These proteins family perform function of cell adhesion, ECM synthesis, collagen fibrillogenesis, proliferation, apoptosis, migration, growth factor production, morphology, and biomeralization. 7

Periostin binds directly to collagen type 1, bone morphogenetic protein (BMP)-1, tenascin C, and fibronectin to maintain integrity and healing of the connective tissue. 8 It is a nonstructural protein that comprises 835 amino acids with a molecular weight of 90 kDa. 7 It was also reported that these are vitamin K–dependent protein. Periostin was initially named as osteoblast-specific factor-2 (OSF-2) as they were first identified in the mouse on gene *MC3T3-E1* of the osteoblastic cell. 6 Later, it was renamed as periostin since they were expressed in periosteum and PDL. Till date, four isoforms of periostin have been identified of which two of them with molecular weight 87 and 90 kDa were found to be originated from fibroblast-derived neuroectoderm. 9 Furthermore, this protein is present not only in periosteum and PDL but also in all collagen-rich tissues such as ligaments, tendon, perichondrium, and cardiac tissue. It was also reported that in adult cardiac tissues, periostin also aid in healing and repair of tissue after pathological conditions. 10 The aim of the present review was to explore the periostin morphology and associated structural features. The periostin immunomodulatory effects and associated biomarkers in context of oral diseases have been discussed.

## Structure and Origin of Periostin

Knowing its origin and from which tissue they are expressed, it is desirable to study its structure and morphology. Structurally, it is originated from *POSTN* gene also called as PN and OSF-2 gene. Currently, eight isoforms of periostin have been discovered. Only four periostin isoforms have been sequenced and elucidated. 11 12 13 Some are classified as larger types and some as specific-tissue type. The more massive types are isoforms of full length expressed by fibroblast and are thought to be associated and secreted in pathological responses. 14 Although the other type is found in physiological responses, they are more stable and are associated in modulating specific tissues such as cell functions regulation, cell to cell interaction, and also found as mechanical sensors. 15

**Fig. 1:**
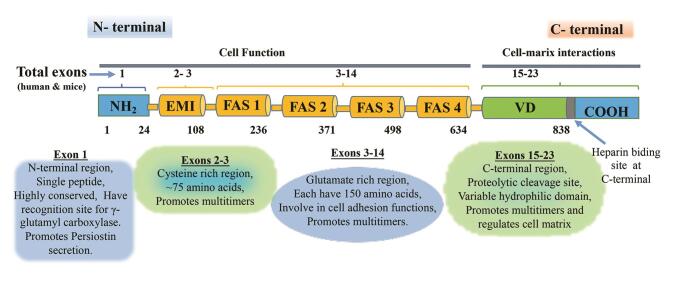
Schematic representation of periostin structural domains. Periostin protein sequence consists of a single sequence, the EMI domain, the four FAS-1 domains, and a variable domain (VD) which comprised nine (15–23) different exons. A combination of exons 15–23 generates four periostin isoforms which have been sequenced and elucidated. Periostin comprised 23 exons. In humans, the gene is located at 13q13.3, but in mice, at 3C. Both in human and mice, the terminal exons are protein coding. The N-terminal region of the periostin promotes cellular functions. The C-terminal binds to extracellular matrix molecular entities and regulates the general organization of the extracellular matrix.

Morphologically, periostin proteins comprise one EMILIN-like (EMI) domain, four repeating tandem of the fasciclin (FAS)-1 domain, and a carboxyl-terminal which has heparin binding site ( Fig. 1 ). 5 EMI domain that belongs to EMILIN family contains amino terminal that is enriched with repeating units of small cysteine domain. Hence, this domain aids in adhesion and interaction with other proteins such as type-1 collagen, tenascin, BMP-1, and fibronectin. 16 Moreover, the different domains belong to fasciclin family and constitute 150 amino acids which are not related to each other. They found to interact with bone morphogenetic factor (BMP) domain. Therefore, as a whole (i.e., EMI domain and FAS-1 domain) promotes collagen cross-linking and enhances the mechanical property of the connective tissue. In humans, FAS-1 domains were also found to guide the growth of axons. This protein not only interacts with ECM and intracellular matrix but also it serves as binding of integrin receptors with ligands, and in this way, they promote cell mobility. 17

## Periostin Immunomodulatory Role in the Oral Cavity

**Fig. 2:**
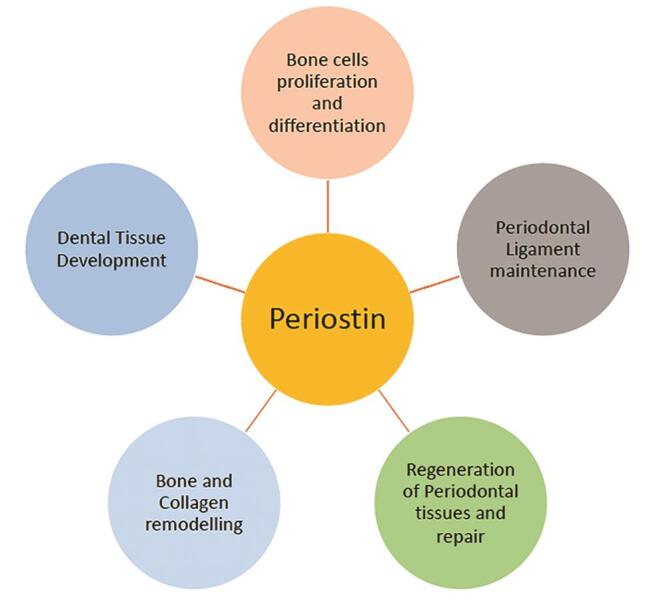
The different functions of periostin in maintaining health of oral and dental tissues.

Periostin contributes to a variety of functions in the physiological performance of various dental tissues including periodontium, gingiva, and alveolus ( Fig. 2 ). PDL, which is a source of large types of cell population and proteins helps in maintaining homeostasis that includes the provision of support to teeth, protection through host defense cells, and provision of sensory signals to masticatory apparatus. Apart from these, PDL cells share significant contribution to wound healing, repair, remodeling, and regeneration of the supporting periodontium. 18 Many reports have shown the significance of periostin in the development of teeth, bones, and cartilage. 19 20 21 It has also been demonstrated that within oral cavity, periostin plays crucial role in maintaining periodontal tissue integrity and bone development. 9 15

Several experimental studies have been reported to find the effect of periostin in both healthy periodontium and diseased tissue processes such as gingivitis, aggressive periodontitis, and chronic periodontitis. 22 Chronic periodontitis affects mostly the adult population, whereas aggressive periodontitis has been shown to be prevalent during adolescence. 22 While assessing the level of periostin within the gingival crevicular fluid (GCF) and in serum of healthy individuals and periodontally compromised patient, it was found that this protein level can be contemplated as a future biomarker to evaluate the disease activity and susceptibility.

Periostin level decreases, when the disease progressed, and the severity of the periodontitis was inversely proportional to periostin level. Clinical studies on aggressive periodontitis and chronic periodontitis revealed distinct salivary and GCF periostin profile. In both conditions, GCF periostin level was significantly reduced, whereas saliva periostin level was elevated in aggressive periodontitis. These data suggest the periostin role in immune response during periodontitis. 22 Different roles of periostin in immunomodulation in various parts of the body have been described ( Table 1 ).

**Table 1:** Role of periostin in Immunomodulation in various tissues

Conditions	Mechanism of periostin in diseases	Function periostin in disease	References
Cardiac repair	Reduction in infarcts size, decrease in fibrosis through improves ventricular remodeling	Development of embryonic heart, expressed in valve leaflets, epicardium, and supporting structures	10 23
Tumorigenesis	Tumor development and metastasis, epithelial–mesenchymal transition. It also promotes lymph-angiogenesis in head and neck cancer	Overexpression in cancers such as lung, colon, breast, head and neck, ovarian, and pancreatic cancer	24 25
Bronchial asthma	Mediates hyperresponsiveness of airway, mucus metaplasia, inflammation, the proliferation of airway fibroblasts in asthma	T-helper type-2 cells produce IL-13 which expresses periostin from the epithelial cells of bronchi of lungs	26
Obesity	Repair and expansion adipose tissue	Expressed in adipose tissue in visceral and subcutaneous depots	15
Diabetes	Homeostatic role in type-2 diabetes	Biomarker for disease progression	27
Pemphigus vulgaris and bullous pemphigoid	Periostin is detected at inflammatory sites of oral and skin diseases	Prominent in the superficial dermis	28
Abbreviation: IL, interleukin.			

In the same way, in blistering autoimmune disease such as pemphigus vulgaris and bullous pemphigoid, tissue-associated macrophages and numbers of CD163+ are detected. During the activation of macrophage immunomodulators such as periostin, interleukin (IL)-4, IL-13, and interferon gamma are primed with monocytes. It is also suggested that periostin is detected at inflammatory sites of oral and skin diseases. Fujimura et al reported the skin and serum samples of pemphigus vulgaris and bullous pemphigoid patients to focus on the immunomodulatory effects of tissue-associated macrophages which include periostin, cytokines, chemokines, and matrix metalloproteinases. In their study, they performed immunohistological staining of periostin in diseased patient’s lesions. Periostin came out to be prominent in superficial dermis of both pemphigus vulgaris and bullous pemphigoid patients. 28 Moreover, skin tumors also show the interaction between periostin and cancer stroma. A study performed by Fujimura et al checked the involvement of tumor-associated macrophages in the skin lesions of mycosis fungoides. For the investigation, they examined the levels of periostin, CD163+, CD206+, IL-4, and macrophages through immunohistological technique. Results revealed the prominence of periostin stromal area of cancer, which hence, suggested that at the early stage of mycosis fungoide, there was the dominance of periostin-stimulated macrophages which was responsible for tumor mass formation, while after the plaque stage of cancer, macrophages were dominant for the maintenance of immunosuppressive cancer environment and which gives the therapeutic target for treatment. The periostin exerts an immunomodulatory effect on tumor-associated macrophages in cancer. Periostin is known to be involved in modulating cell function, including the production of chemokines and proinflammatory cytokines. Zhou et al have reported that glioblastoma stem cells secrete periostin and treatment of glioblastoma multiforme can be improved by targeting the periostin-mediated tumor-associated macrophages recruitment. 29 In another study conducted by Wang et al, they demonstrated the limitations that periostin brings in metastatic colonization in disseminated breast tumor cells. They showed that how periostin promotes at the early stage of breast cancer, and the pulmonary accumulation of myeloid-derived suppressor cells which in turn indicates the potentiality of periostin for the prevention and treatment of breast tumor metastasis. 30

During the embryological stage of tooth development, periostin proteins along with collagen have proven to support palatogenesis process while differentiating soft palate and hard palate through transforming growth factor (TGF)-β pathway. 5 Many experimental studies have been performed on mice to study the effect of periostin both embryologically and in the adult stage. Also, it was found that protein not only effective in maintaining normal homeostasis of the periodontium but also plays roles in the development of tooth germ. 31 Moreover, histological analysis showed periostin’s role in postnatal mineralization and tooth formation which was directly linked to mechanical loading. 32 Also, these matricellular adhesive proteins are involved in regions where teeth subjected to mechanical stress or occlusal forces. Hence, these findings can be implicated in humans and should be considered as a means for maintaining tissue health while undergoing any pathological changes or under mechanical stress and during the eruption of tooth. A recent study was done on the human tooth which showed increased expression of periostin within Sharpey’s fiber of molar tooth. Interestingly, the data suggest that periostin proteins can be useful markers during orthodontic tooth movement as their expression is regulated at pressure site. 33

Dental implant is one of the most important fields of dentistry where the outcome is unpredictable due to peri-implantitis. 34 35 36 Evaluation of peri-implant sulcular fluid can be considered as a diagnostic tool for demonstrating health prognosis of peri-implant tissue. 37 Histological studies revealed that periostin protein molecules along with collagen product could be used as a useful marker in detecting inflamed and non-inflamed health status of the implant and its surrounding tissue as they are involved in healing, remodeling and related with response to external forces. This early detection of the health status of implant tissue can help in better prognosis and management of peri-implantitis. 37

## Conclusion

Periostin, categorized as a class of ECM protein, is not only gaining interest in the field of dentistry but also in other areas due to its expression in other tissues of the body. In dental science, this protein can be used in future as a useful tool in the diagnosis of a periodontal disease which can eventually aid in management if early diagnosis is made through this biomarker. 38 39 Furthermore, this protein can also be used in the field of orthodontic as the expression of this protein is modulated when external forces are applied. 33 Hence, treatment prognosis can better be visualized through this protein analysis. Also, periostin can contribute as a local factor in bone and periodontal remodeling during orthodontic tooth movement. 14

As described earlier, periostin molecules are also found in heart tissue. According to known data, it is thought to be a promising therapeutic protein in myocardial infarction that can play a crucial role in healing and regeneration of cardiac tissue. 40 Bronchial asthma that is a chronic pathological inflammatory response was caused because of Th-2 type immune cells. Histological evidence showed that periostin protein involved in fibrosis of airways epithelial cells by activating TGF-β, IL-4, IL-3, and other inflammatory mediators. 15 Cancer is one of the critical causes of worldwide mortality that is being treated by surgical technique, chemotherapy, and radiotherapy. Scientists have been working to find specific biomarkers that can help in management and early diagnosis of the metastasized tumor to control death ratio through this disease. 41 In cancer biology, studies showed that this protein molecule could be used as diagnostic biomarkers in tumor metastasis, but further investigations are needed. 15
